# Neuroprotective Effects of Anthraquinones from Rhubarb in Central Nervous System Diseases

**DOI:** 10.1155/2019/3790728

**Published:** 2019-05-16

**Authors:** Xun Li, Shifeng Chu, Yinjiao Liu, Naihong Chen

**Affiliations:** ^1^College of Pharmacy, Hunan University of Chinese Medicine, Changsha 410208, China; ^2^Institute of Materia Medica, Chinese Academy of Medical Sciences and Peking Union Medical College, Beijing 100050, China

## Abstract

Rhubarb is a well-known traditional Chinese medicine; it has been used in China for thousands of years. Rhubarb anthraquinones are the major medicinal ingredients derived from rhubarb including emodin, aloe-emodin, chrysophanol, rhein, physcion, and danthron. These different anthraquinone derivatives alone or in combination play a therapeutic role in central nervous system diseases (CNSD), such as cerebral ischemic stroke, intracerebral hemorrhage, traumatic brain injury, brain tumor, Alzheimer's disease, depression, and others. We review the experimental studies on these six anthraquinones in the treatment of CNSD by consulting literature published in the last 20 years in PubMed and then give a future perspective on it. In the end of this paper some deficiencies related to these studies also have been pointed out.

## 1. Introduction

**Figure 1:**
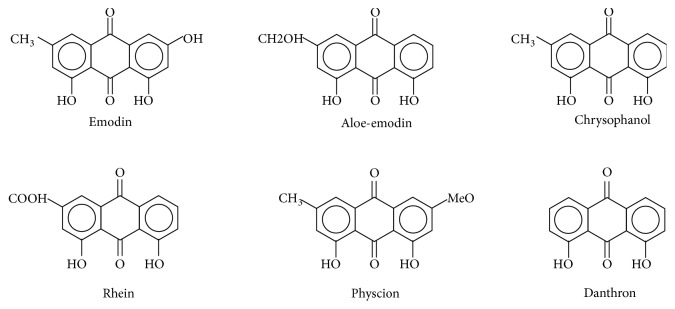
Chemical structures of anthraquinone derivatives in rhubarb.

Rhubarb (Da Huang) is dried rhizomes and roots of* Rhubarb palmatum* L,* Rheum tangguticum *Maxim. ex Balf, or* Rheum officinale *Baill from Polygonaceae family [1]. It is one of the oldest and most widely known Chinese herbal medicines, first reported in Shen Nong's Materia Medica from Han Dynasty [2]. Rhubarb is normally used as an effective laxative, and many Chinese herbal prescriptions contain it. The main effective ingredients of rhubarb are anthraquinone derivatives including emodin (1,3,8-trihydroxy-6-methylanthraquinone, 2.6%), aloe-emodin (1,8-dihydroxy-3-hydroxyl-methylanthraquinone, 1.8%), chrysophanol (1,8-dihydroxy-3-methyl-anthraquinone, 1.9%), rhein (1,8-dihydroxy-3-carboxyanthraquinone, 1.9%), physcion (1,8-dihydroxy-3-methyl-6-methoxy anthraquinone, 0.8%), and danthron (1,8-dihydroxy-9,10-anthraquinone, <0.2%) (Figure 1) [3]. These anthraquinone derivatives are also used as the quality control standard of rhubarb according to the Chinese Pharmacopoeia [4, 5]. Modern pharmacological studies indicated that these anthraquinones possess a wide spectrum of pharmacological properties, such as anti-inflammatory, antioxidant, antitumor, and antivirus [6–11].

Cerebral ischemic stroke (CIS), intracerebral hemorrhage (ICH), traumatic brain injury (TBI), brain tumor, Alzheimer's disease (AD), and depression are common diseases of central nervous system. Patients with these diseases usually end up with death or disability, and the treatment of these diseases consumes huge amounts of social wealth [12–16]. Chinese herbal medicines have attracted widespread attention because it has the lower risk and less cost than many other conventional treatments [17]. In recent years, emerging experimental researches about these anthraquinones in the treatment of central nervous system diseases (CNSD) have been done, but there are no relevant reviews which have been published so far. So the purpose of this review is to give a comprehensive summary and analysis of various major anthraquinones from rhubarb in the treatment of CNSD during the past two decades.

## 2. Neuroprotective Effects of Anthraquinones from Rhubarb in Different CNSD

### 2.1. Cerebral Ischemic Stroke

The occlusion of vascular and blockage of cerebral blood flow, brain ischemia and hypoxia, and necrosis of brain tissue are commonly caused by CIS; these results eventually lead to neurological dysfunction [18]. Neurobiochemical and molecular biological mechanisms lead to neuronal damage in CIS including postischemic inflammation, oxidative stress, reperfusion injury, neuronal apoptosis, excitotoxicity, blood-brain barrier (BBB) dysfunction, microvascular injury, etc. [19, 20]. Rhubarb anthraquinones which play a neuroprotective role in CIS are mainly emodin, chrysophanol, and rhein, or different rhubarb anthraquinones act together. Different anthraquinones with the relevant mechanisms will be introduced in four parts as follows.

Firstly, emodin exerts neuroprotective effects in CIS by maintaining the integrity of BBB, reducing inflammation and inhibiting apoptosis [18, 21–24]. Yan Li et al. [18] found that emodin improved the neurological deficit scores (NDS), reduced BBB permeability, and decreased the infarction area in cerebral ischemia/reperfusion (I/R) model rats; these results were probably due to the inhibitory effect of emodin against the expressions of connexin 43 (Cx43) and aquaporin 4 (AQP4). Cx43 belongs to a member of membrane protein family, it composes the basic structure and function of intercellular gap junctions [25], and AQP4 is a kind of membrane water channel protein that plays an important role in cerebral edema and brain water balance [26]. In another* in vivo* study, emodin inhibited cascade inflammatory reaction by increasing the level of growth transforming factor-*β* (TGF-*β*) and depressing the levels of tumor necrosis factor-*α* (TNF-*α*), interleukin-1*β* (IL-1*β*), and intercellular cell adhesion molecule-1 (ICAM-1) and thus improved the neurological symptom evaluation score, brain water ratio, and cerebral infarction area in model rats [21]. On the other hand, a number of* in vitro* studies have demonstrated that emodin could inhibit neuronal apoptosis [22, 23, 27]. Sung Min Ahn et al. [22] reported that emodin reduced glutamate-induced apoptosis by increasing B-cell lymphoma-2 (Bcl-2) expression and decreasing the expressions of caspase-3 and Bax in HT22 cells; what is more, it also increased the expression of mature brain-derived neurotrophic factor (BDNF) and the phosphorylations of Akt and cAMP response element binding protein (CREB) and therefore improved behavioral function in photothrombotic ischemic mice. Similarly, Liu T et al. [23] suggested that emodin inhibited hydrogen peroxide (H_2_O_2_)-induced apoptosis in primary rat cortical neurons. Besides, it has been found that emodin could inhibit neuronal apoptosis and alleviate the injury of PC12 nerve cells after oxygen-glucose deprivation via increasing the expression of activin A[24], which belongs to transforming growth factor *β*1 signal transduction system superfamily and exerts neuroprotective effect[27, 28].

Secondly, the neuroprotective mechanisms of chrysophanol (CHR) in CIS are mainly associated with antinitrosative/oxidative, anti-inflammatory, and antiapoptosis [29–31]. Yongmei Zhao et al. [29] suggested that CHR could suppress NO-associated neuronal cell death through attenuating the levels of nitrite plus nitrate (NO_x_^−^) and 3-nitrotyrosine (3-NT) and decrease cleaved caspase-3 protein expression. Besides, CHR increased the total superoxide dismutase (SOD) and manganese-dependent SOD (MnSOD) activities and inhibited reactive oxygen species (ROS) generation in the cerebral I/R model. Meanwhile, Nan Zhang et al. [30] reported that CHR inhibited inflammatory response via reducing the expressions of IL-1*β*, caspase-1 as well as NALP3 and then decreased neurological deficits, infarct volume, brain edema, and BBB permeability in I/R mice. NALP3 belongs to NALP3 inflammasome consisted of NACHT domain-, leucine-rich repeat-, and pyrin domain-containing protein 3 (NALP3), the adaptor protein apoptosis-associated speck-like protein (ASC), and caspase-1, and the function of it is to activate IL-1*β* then triggers inflammatory cascade [32, 33]. Similarly, CHR also exhibited anti-inflammatory actions by attenuating the expressions of TNF-*α*, IL-1*β*, and NF-*κ*B p65 and finally improved the survival rate, neurological assessment, and motor function in middle cerebral artery occlusion mice [34]. What is more, CHR could inhibit inflammatory response and neuronal apoptosis in I/R injury mice by attenuating the expression of endoplasmic reticulum (ER) stress-related factors including glucose-regulated protein 78 (GRP78), phosphorylated eukaryotic initiation factor 2*α* (p-eIF2*α*), CCAAT-enhancer-binding protein homologous protein (CHOP), and caspase-12 as well as inhibitory *κ*B-*α* (I*κ*B-*α*) and the inhibitor of NF-*κ*B [31].

Thirdly, QIPENG ZHAO et al. [35] reported that rhein exhibited neuroprotective effects by inhibiting oxidative stress and apoptosis. Rhein could decrease malondialdehyde (MDA) level and enhance the activities of SOD, catalase (CAT), and glutathione peroxidase (GSH-Px). Moreover, rhein markedly reduced the expression of BAX, caspase-9, caspase-3, and cleaved caspase-3, meanwhile, increased the expression of Bcl-2, then improved neurological functional scores (NFSs), and reduced infarction area in I/R injury rats.

Fourthly, a number of studies have indicated that different rhubarb anthraquinones act together in the treatment of CIS. Qinxiao Guan et al. [36] found that optimized rhubarb aglycone (aloe-emodin: rhein: emodin: chrysophanol:physcion = 50:76:38:105:68) regulated amino acid, energy and lipid metabolic disturbance related to the I/R injury by increasing the plasma levels of lipids, phosphocreatine/creatine, and the urine levels of taurine, tyrosine, *α*-ketoglutaric acid/creatinine, and decreasing the plasma levels of lactate, taurine, glutamate, glycine, methionine/glucose, and the urine levels of choline, glycine, and proline/glucose. Accordingly, rhubarb anthraquinones could alleviate neurological impairment and cerebral infarction area and inhibit neuronal apoptosis in I/R rats. Meanwhile, Li j et al. [37] reported that rhubarb aglycone reduced thrombolysis-caused brain microvascular basement membrane impairment through decreasing the level of IgG and increasing the level of type IV collagen (CoLIV) and laminin (LN) and then decreased the intracranial hemorrhage ratio and mortality in cerebral ischemia rats. What is more, Xiangping Lin et al. [38] found 287 differentially expression proteins (DEPs) and 76 overlapping DEPs between model group (MG) versus sham group (SG) and rhubarb group (RG) versus MG with iTRAQ-based proteomics analysis. There were 14 DEPs related pathways including synaptic vesicle cycle, cGMP-PKG signaling pathway, amyotrophic lateral sclerosis, long-term potentiation, tuberculosis, and so on. 76 overlapping DEPs are mainly associated with synaptic vesicle cycling. Furthermore, compared with the MG, rhubarb treatment significantly increased the protein expression of Syn1 and ERK1/2, which are pathway proteins related to synaptic transmission and plasticity [39, 40]. Interestingly, a pharmacokinetics study showed that the model group's maximum plasma concentration (C_max_), half-life (t_1/2_), and area under curve (AUC_0-t_) of aloe-emodin, rhein, emodin, and chrysophanol were remarkably increased, but the total body clearance (CL) values were significantly decreased compared with normal group. These results demonstrated anthraquinones were easier to be absorbed in CIS condition [41].

### 2.2. Intracerebral Hemorrhage

Plenty of studies have suggested that rhubarb anthraquinones exhibit protective effects in ICH and its underlying mechanisms of action are involved in anti-inflammation, antiapoptosis, and protection of BBB [42–44]. It has been reported that emodin triggered microglia cell apoptosis by increasing the activity of caspase 3/7 and promoted activated microglia cell apoptosis through reducing the level of TNF-*α* and IL-1*β*. Meanwhile, emodin induced the expression of TRB3 during microglial apoptosis, which is a proapoptotic gene associated with oxidative stress and apoptosis [45, 46]. Moreover, overexpression of TRB3 induced microglial cell apoptosis and knockdown of TRB3 attenuated the apoptotic effect of emodin [42]. On the other hand, YANG WANG et al. [43] found that rhubarb or its active components (aloe-emodin, rhein, emodin, and chrysophanol) relieved neurological symptoms and attenuated BBB permeability by enhancing zonula occludens-1 (ZO-1) expression in ICH rats. ZO-1 is a main kind of tight junction proteins, which constitutes an important part of BBB [47]. Similarly, Tang YP et al. [44] suggested rhubarb could maintain BBB integrity and reduce astrocyte end feet process swelling by inhibiting the expression of AQP-4 gene.

### 2.3. Traumatic Brain Injury

Many of studies have proved that rhubarb anthraquinones exert therapeutic effects in TBI and its associated mechanisms including anti-inflammation, antiglutamate excitotoxicity, antioxidative stress, and protection of the BBB integrity [48–53]. Yuan Ma et al. [48] found that emodin reduced brain damage and improved behavioral observation by inhibiting the expression and activity of inducible nitric oxide synthase (iNOS) and then consequently attenuated the generation of NO after blast-induced TBI in mice. Interestingly, Jian-Wen Gu et al. [49] reported that emodin inhibited the excitatory postsynaptic potential (EPSP) and markedly improved paired-pulse facilitation (PPF) of the EPSP on CA1 pyramidal neurons. Furthermore, the inhibition of the EPSP induced by emodin was blocked by either adenosine deaminase or 8-CPT, an adenosine A1 receptor antagonist, whereas the inhibitory synaptic transmission was not affected by emodin. The results suggested rhubarb extracts exerted neuroprotective effects by inhibiting glutamate excitotoxicity. Additionally, Lin XZ et al. [54] reported that emodin and rhubarb polysaccharides played the opposite role in brain intracellular Ca^2+^ level.

On the other hand, Yang Wang and his colleagues [50–52] reported that rhubarb and rhein exhibited protective effects after TBI through inhibiting extracellular regulated kinase (ERK)/matrix metalloproteinase-9 (MMP-9) pathway and reducing oxidative stress; besides, they could reduce brain edema and BBB permeability through inhibiting ERK/MMP-9 pathway by preventing activation of gp91phox subunit of NADPH oxidase induced ROS production* in vitro* and* in vivo *[50]. Similarly, another study suggested that oral administration of rhubarb downregulated MMP-9 and upregulated ZO-1 by inhibiting ERK signaling pathway [51]. What is more, rhubarb and rhein also improved the activities of SOD and CAT, increased the level of glutathione (GSH) and the ratio of GSH/glutathione disulfide (GSSG) and decreased the levels of MDA and GSSG in TBI rats [52]. In the end, Wang ZP et al. [53] reported that rheum tanguticum polysaccharides could decrease water content and the level of MDA and improve the activities of Na+-K+ ATPase and total SOD.

### 2.4. Brain Tumor

The antitumor effects of rhubarb anthraquinones are mainly focused on the inhibition of brain tumor growth by inducing apoptosis[10, 55–58]. Plenty of* in vitro* studies have shown danthron can inhibit glioma growth[10, 55–57], which is a brain tumor with poor prognosis and usually develops into high-grade malignancies[15, 59]. Danthron was reported to induce C6 rat glioma cells apoptosis via ROS-associated and mitochondria-mediated pathways, it reduced mitochondrial membrane potential level, released cytochrome c, apoptosis-inducing factor (AIF), and endonuclease G (Endo G) from mitochondria and increased the levels of caspase-9/3; meanwhile, it also increased the production of ROS and this effect could be reversed by ROS scavenger N-acetyl-L-cysteine[10].CHIN-CHUNG LIN et al. [55] suggested that danthron inhibited the invasion and migration of glioblastoma multiforme GBM 8401 cells via decreasing the expressions of focal adhesion kinase (FAK), MMP-7, MMP-9, uPA, and Rho-associated kinase 1 (ROCK-1). Glioblastoma is one of the most aggressive and malignant forms of glioma [60]. Similarly, Hsu-Feng Lu et al. [56] found that danthron killed and induced apoptosis of GBM 8401 cells in concentration- and time-dependent manner. The potential mechanism might relate to increasing the levels of ROS, cytosolic Ca^2+^, caspase-8/9, and Bax, decreasing the levels of mitochondrial membrane potential and pro-caspase-8/9 proteins, and activating caspase-3/8/9. Besides, the inhibitors of caspase-3/8/9 blocked the activation effect of danthron against these factors. Moreover, the same author reported that danthron induced DNA damage in GBM 8401 cells via decreasing the expression of DNA damage and repair genes such as ataxia-telangiectasia mutated (ATM), ataxia-telangiectasia and Rad3-related (ATR), breast cancer 1, early onset (BRCA-1), 14-3-3 proteins sigma (14-3-3*σ*), DNA-dependent serine/threonine protein kinase (DNA-PK), and* O*^*6*^-methylguanine-DNA methyltransferase (MGMT) [57]. On the other hand, aloe-emodin (AE) was reported to inhibited U87MG human glioma cells growth and block U87MG in S and G2/M phase via increasing the protein levels of p53/p21 and decreasing the phosphorylation of AKT as well as inhibiting the expression of CDK2, which is an active protein in the S phase of the cell cycle. Meanwhile AE also induced U87MG apoptosis by reducing the expression of poly (ADP-ribose) polymerase (PARP) and activating Lamin A. In addition, AE markedly reduced the U87MG cell density and tumor size and improved the levels of P53 and caspase 8/3* in vivo *[58]. Physcion has been reported to increase the expression of *α*2,8-sialyltransferase (hST8Sia VI) gene through ERK and p38 MAPK pathways in SK-N-BE(2)-C human neuroblastoma cells[11]. HST8Sia VI gene is a kind of human sialyltransferase gene, which plays an important part in cell differentiation/adhesion and malignant transformation [61].

### 2.5. Alzheimer's Disease

AD mainly causes cognitive impairment in the elderly. The neuropathological hallmarks of AD are senile plaques and neurofibrillary tangles containing *β*-amyloid protein (A*β*) [62]. Four of rhubarb anthraquinones (emodin, CHR, rhein, and danthron) exhibit therapeutic effects in AD. First, Tao Liu et al. [63] reported that emodin markedly decreased the cortical neuron's death induced by A*β*_25-35_, and this effect was blocked by PI3K pathway inhibitor LY294002 or estrogen receptor antagonist ICI182780, but not by U0126, which is an inhibitor of ERK. Besides, emodin increased Bcl-2 expression and the phosphorylation of Akt, and decreased the levels of phospho-Jun-N-terminal kinases (JNK) 1/2. At the same time, Yan-ping Sun et al. [64] suggested that emodin markedly reduced the level of lactate dehydrogenase and inhibited cell viability as well as suppressed the conversion ratio of LC3-I/LC3-II in A*β*PP/PS1 mice and PC12 cells, which has been considered to play a significant role in autophagy and eventually result in cell death [65]. Moreover, emodin significantly reduced the level of LC3-II positive cells in the cortex of A*β*PP/PS1 mice, improved the expression of Bcl-2, and reduced the expressions of Beclin-1 and the class III PI3K (hVps34) induced by A*β*_25-35_, of which (Beclin-1/hVps34) pathways can promote LC3-I transform to LC3-II [66]. Interestingly, Peng Zeng et al. [67] found that emodin decreased A*β* levels and tau hyperphosphorylation via reducing *β*-site amyloid precursor protein-cleaving enzyme 1 (BACE 1) levels and increasing protein phosphatase 2A (PP2A) activity in AD-like rats' hippocampal; meanwhile, it also increased hippocampal neuron numbers and synapse-related proteins, suppressed oxidative stress by regulating MDA/SOD, and decreased DNA methyltransferases 1/3*β* levels, which are associated with DNA methylation impairments; moreover, emodin inhibited microglial activation through reducing 5-lipoxygenase (5-LO), IL-6, and TNF-*α* levels and then improved cognitive function and cerebral microvascular integrity in AD-like rats.

Second, Unbin Chae et al. [68] reported that CHR increased the viability of neuronal cells induced by glutamate in a dose-dependent manner; meanwhile, it inhibited neuronal apoptosis via increasing Bcl-2 expression and decreasing the expressions of Bax and AIF. In addition, CHR reduced ROS levels and prevented mitochondrial fission by suppressing the dephosphorylation of dynamin-related protein 1 (Drp 1) in hippocampal, which is one of the major regions suffered from excessive cell death in AD.

Third, Jiang Liu et al. [69] reported that rhein lysinate, an active component of Rheum tanguticum Maxim, significantly reduced the *β*-Amyloid precursor protein (APP) of AD model rats by improving the expression of sirtuin 1 (SIRT1); it also decreased the expression of TNF-*α* and IL-6, reduced the levels of ROS, and increased the levels of glutathione peroxidase (GSH-px) and SOD in AD rats. Interestingly, two kinds of rhein hybrids have been synthesized as a potential anti-Alzheimer drug candidate [70–73]. One of them is rhein-huprine hybrids; it could alleviate the A*β*-induced synaptic dysfunction, increase the content of synaptic proteins, and induce the long-term potentiation (LTP) in brain slices of C57bl6 mice; meanwhile, it also reduced the levels of A*β* and improved the levels of mature APP in APP-PS1 transgenic mice. What is more, rhein-huprine hybrids suppressed the activities of human acetylcholinesterase (AChE) and butyrylcholinesterase (BuChE) as well as *β*-secretase (BACE-1), of which (BACE-1) function is to promote the synthesis of APP to A*β* [74, 75] and reduce the aggregation of A*β* in vitro [70]. The latest study reported that rhein-huprine hybrids decreased A*β* levels and memory disorders, induced LTP, and reduced tau phosphorylation and brain inflammation in AD or AD-like rats [72]. Furthermore, the modified rhein-huprine hybrids also suppressed the activities of AChE and BACE-1 via reducing oxidative stress and A*β*/tau aggregation [73]. The other one is tacrine-rhein hybrids; research indicated that this hybrid could inhibit AChE-induced A*β* aggregation; meanwhile, it also had metal-chelating activity with less side effects [71].

Fourth, danthron has been reported to inhibit neuronal injury induced by A*β* with a concentration-dependent manner; it also suppressed Fe^31+^-induced oxidative damage via reducing membrane lipid peroxidation in primary cortical cells. What is more, it reduced the damage of neuronal cells induced by sodium nitroprusside and H_2_O_2_ as well as buthionine sulfoximine, an inhibitor of endogenous GSH synthesis [62].

### 2.6. Depression and Other Nervous System Diseases

Depression is a common and complicated disease; the physiopathology of depression is associated with plenty of bioprocesses including the deficiency of monoamine neurotransmitter activity, the overactivity of hypothalamic-pituitary-adrenal axis (HPA axis), and the inflammation and neurodegeneration[16, 76]. Meng Li et al. [77] reported that emodin could reduce serum corticosterone level and improve behavioral performance on depression mice; besides, it also increased the expression of BDNF and glucocorticoid receptor (GR) and relieved the anhedonia symptom by increasing sucrose consumption in depression mice. In addition, Kai Zhang et al. [78] suggested that CHR alleviated the inflammation of LPS-induced depression rats via reducing the levels of IL-1*β*/6 and TNF-*α* and attenuated the expression of P2X7/NF-*κ*B pathway related proteins including P2X7, p-IKK*α*, p-IKK*β*, p-I*κ*B*α*, and p-NF-*κ*Bp65.

Tao Yang et al. [79] reported that emodin could restore the behavioral disorders and abnormal electroencephalogram (EEG) changes in epileptic rats by attenuating the expressions of multidrug resistance gene 1 (MDR1), cyclooxygenase-2 (COX-2), N-methyl-D-aspartate (NMDA) receptor, and P-glycoprotein [79–83]. At the same time, emodin also has been demonstrated to alleviate the hyperalgesia of chronic constriction injury rats via reducing the expression of P2X_2/3_ receptor, which acts an important part in neuropathic pain [84, 85]. On the other hand, CHR has been reported to improve cognition function by decreasing the expressions of IL-1*β*/4/6 and TNF-*α*; and it also reduced nerve cell death via inhibiting astrocyte activities in diabetic mice[8]. Besides, CHR reduced the learning and memory damage of lead-exposed rats and improved lead-induced mitochondria and rough endoplasmic reticulum injury in hippocampal neurons and capillary endothelial cells. The potential mechanism of this protective effect might relate to increasing the activities of SOD and GSH-Px and decreasing the level of MDA [86]. Moreover, Shu-Jen Chang et al. [87] suggested that rheum palmatum methanol extract, which contains higher levels of emodin, aloe-emodin, chrysophanol, rhein, and physcion than water extract, could kill Japanese encephalitis virus (JEV) and inhibit JEV yields and infectivity.

## 3. Conclusion

Anthraquinones are major bioactive ingredients founded in rhubarb. Although some other Chinese medicinal herbs also contain it, such as* Polygonum multiflorum *Thunb [22] and* Rhizoma Polygoni Cuspidati [24], *most of the studies focus on these six anthraquinones (emodin, aloe-emodin, CHR, rhein, physcion, and danthron) derived from rhubarb. Emodin, aloe-emodin, and CHR mainly show neuroprotective effects by anti-inflammation, antioxidant stress, and maintaining the integrity of BBB in CNSD [8, 18, 21–24, 29–31, 34, 42, 48–50, 52, 63, 67–69, 78, 86]. Interestingly, emodin showed contradictory effects on cell apoptosis. Many of studies indicated emodin could inhibit neuronal apoptosis in CIS [22–24], whereas Xueping Zhou et al. [42] reported that emodin induces microglial cell apoptosis in ICH. Moreover, aloe-emodin also induced glioblastoma cell apoptosis [58]. Studies on the neuroprotective effects of rhein are mainly in TBI and AD, the mechanism of which is associated with anti-inflammation and antioxidant stress [50, 52]. Danthron basically plays an antitumor role in CNSD [10, 55–57]. From all the* in vitro* and* in vivo* experiments currently searchable (Table 1), these bioactive anthraquinones have convincingly demonstrated neuroprotective activities in CNSD and the related pharmacological pathway involving multiple cellular and molecular targets, which indicates these anthraquinones have a broad therapeutic potential in the future.

Most of the studies on the neuroprotective effects of anthraquinone compounds from rhubarb have focused on anthraquinone monomers. The molecular structures of different anthraquinone monomers are very similar (Figure 1); for example, CHR and rhein almost have same molecular structure, except one of CHR's methyl groups is substituted by rhein's carboxyl group; they both inhibited the expression of cleave caspase-3 and attenuated the activity of SOD in CIS [29, 35]; however, which effects are stronger is still unknown. The same as emodin and CHR, emodin removes one of hydroxyl group then become CHR; they both demonstrated anti-inflammatory properties in CIS by downregulating the expression of TNF-*α* and IL-1*β* [21, 30, 34], while which ingredient works better remains obscure. There are many other studies reported that anthraquinones or rhubarb has neuroprotective activities as a whole, but whether/how the monomer components interact with each other remains unclear. Therefore, further researches not only need to be carried out in order to explore more neuroprotective mechanisms of rhubarb anthraquinones in CNSD, but also to find out which anthraquinones monomers have the best therapeutic effects and whether/how they could interact with each other.

## Figures and Tables

**Table 1:** List of the neuroprotective effects of anthraquinones in CNSD.

Disease	Anthraquinones	Outcomes	Application	Ref.
Ischemic stroke	Emodin	Improves NDS, reduces BBB permeability and decreases the infarction area via inhibiting the expression of Cx43 and AQP4	*In vivo*	18

	Emodin	Improves neurologic symptom evaluation score, brain water ratio and cerebral infarction area; inhibits cascade inflammatory reaction by increasing TGF-*β* level and depressing the levels of TNF-*α*/IL-1*β*/ICAM-1	*In vivo*	21

	Emodin	Enhances behavioral function, against apoptosis through increasing Bcl-2 expression and decreasing caspase-3 and BAX expression; increases BDNF expression and the phosphorylation of Akt/CREB	*In vitro*and *In vivo*	22

	Emodin	Inhibits H_2_O_2_-induced apoptosis in primary rat cortical neurons	*In vitro*	23

	Emodin	Inhibits neuronal apoptosis and alleviates the injury of PC12 nerve cells via increasing the expression of activin A	*In vitro*	24

	Chrysophanol	Inhibits NO-induced neuronal death through attenuating the levels of NO_x_^−^/3-NT, and decreases cleaved caspase-3 expression; increases total SOD/MnSOD activities and inhibits ROS generation	*In vivo*	29

	Chrysophanol	Decreases neurological deficits, infarct volume, brain edema and BBB permeability; inhibits inflammatory response via reducing the expression of IL-1*β*, caspase-1 as well as NALP3	*In vivo*	30

	Chrysophanol	Improves survival rate, reduces brain tissue loss, and improves neurological assessment and motor function; reduces the expression of TNF-*α*, IL-1*β* and NF-*κ*B p65	*In vivo*	34

	Chrysophanol	Inhibits inflammatory response and neuronal apoptosis by attenuating the expression of ER stress-related factors including GRP78, CHOP, caspase-12 and I*κ*B-*α*	*In vivo*	31

	Rhein	Improves NFSs and reduces infarction area; decreases MDA level and enhances the activities of SOD/CAT/GSH-Px; reduces the expression of BAX, caspase-9/3 and cleaved caspase-3, and increases the expression of Bcl-2	*In vivo*	35

	Rhubarb anthraquinones	Alleviates neurological impairment and cerebral infarction area, and inhibits neuronal apoptosis; increases the plasma levels of lipids, phosphocreatine/creatine and the urine levels of taurine, tyrosine, *α*-ketoglutaric acid/creatinine, and decreases the plasma levels of lactate, taurine, glutamate, glycine, methionine/glucose and the urine levels of choline, glycine, proline/glucose	*In vivo*	36

	Rhubarb anthraquinones	Decreases the intracranial hemorrhage ratio and mortality; reduces brain microvascular basement membrane impairment through decreasing the level of IgG and increasing the levels of CoLIV/LN	*In vivo*	37

	Rhubarb	Finds 287 DEPs and 76 overlapping DEPs between MG versus SG and RG versus MG; the related pathways including synaptic vesicle cycle, cGMP-PKG signaling pathway, amyotrophic lateral sclerosis, long-term potentiation, tuberculosis, and so on; increases the protein expression of Syn1 and ERK1/2	*In vivo*	38

	Rhubarb anthraquinones	The C_max_, t_1/2_ and AUC_0−t_ of aloe-emodin, rhein, emodin and chrysophanol are increased in CIS condition	*In vivo*	41

Intracerebral hemorrhage	Emodin	Triggers microglia cell apoptosis by increasing the activity of caspase 3/7; promotes activated microglia cell apoptosis by reducing the level of TNF-*α* and IL-1*β*, and induces the expression of TRB3 during microglial apoptosis	*In vitro*	42

	Rhubarb or rhubarb anthraquinones	Relieves neurological symptoms and attenuates BBB permeability by enhancing ZO-1 expression	*In vivo*	43

	Rhubarb	Maintains BBB integrity and reduces astrocyte end feet process swelling by inhibiting AQP-4 expression	*In vivo*	44

Traumatic brain injury	Emodin	Reduces brain damage, improves behavioral observation by inhibiting the expression and activity of iNOS, and attenuates the generation of NO	*In vivo*	48

	Emodin	Inhibits the EPSP, improves PPF of the EPSP, and inhibits glutamate excitotoxicity	*In vitro*	49

	Rhein and rhubarb	Reduces brain edema and BBB permeability through preventing activation of gp91phox subunit of NADPH oxidase induced ROS production; inhibits ERK/MMP-9 pathway	*in vivo* and* in vitro*	50

	Rhubarb	Down-regulates MMP-9 and up-regulates ZO-1 by inhibiting ERK signaling pathway	*in vivo *	51

	Rhein and rhubarb	Improves the activities of SOD and CAT, increases the level of GSH and the ratio of GSH/GSSG, and decreases the levels of MDA and GSSG	*in vivo *	52

	Rheum tanguticum polysaccharides	Decreases water content and the level of MDA, and improves the activities of Na+-K+ ATPase and total SOD	*in vivo*	53

	Emodin	Regulates the brain intracellular Ca^2+^ level	*In vitro*	54

Brain tumor	Danthron	Reduces mitochondrial membrane potential level, releases cytochrome c, AIF and Endo G from mitochondria, and increases caspase-9/3 levels and ROS production	*in vitro*	55

	Danthron	Inhibits the invasion and migration of GBM 8401 via decreasing the expressions of FAK, MMP-7, MMP-9, uPA and ROCK-1	*in vitro*	56

	Danthron	Kills GBM 8401 cells and induces apoptosis by increasing the levels of ROS, cytosolic Ca^2+^, caspase-8/9 and Bax, decreasing mitochondrial membrane potential levels and pro-caspase-8/9 protein levels, and activating caspase-3/8/9	*in vitro*	57

	Danthron	Induces DNA damage in GBM 8401 cells via decreasing the expressions of DNA damage and repair genes such as ATM, ATR, BRCA-1, 14-3-3*σ*, DNA-PK and MGMT	*in vitro*	58

	Aloe-emodin	Inhibits U87MG human glioma cells growth and blocks U87MG in S and G2/M phase via increasing the protein level of p53/p21 and decreasing AKT phosphorylation and CDK2 expression; induces U87MG apoptosis by reducing PARP expression and activating Lamin A, reduces U87MG cell density and tumor size, and improves the levels of P53 and caspase 8/3	*in vivo *and* in vitro*	59

	Physcion	Increases the expression of hST8Sia VI through ERK and p38 MAPK pathways in SK-N-BE(2)-C human neuroblastoma cells	*in vitro*	63

Alzheimer's disease	Emodin	Decreases A*β*_25-35_-induced neuron death and the levels of phospho-JNK 1/2, increases the phosphorylation of Akt and Bcl-2 expression	*in vitro*	66

	Emodin	Inhibits cell viability and the conversion ratio of LC3-I/LC3-II, reduces the level of lactate dehydrogenase; decreases the level of LC3-II positive cells in the cortex of A*β*PP/PS1 mice; improves Bcl-2 expression and reduces the expressions of Beclin-1 and hVps34	*in vivo *and *in vitro*	67

	Emodin	Decreases A*β* aggregation and tau hyperphosphorylation via reducing BACE1 levels and increasing PP2A activity; increases hippocampal neuron numbers and synapse-related proteins; suppresses oxidative stress by down-regulating MDA and up-regulating SOD, decreases DNA methyltransferases 1/3*β* level, inhibits microglial activation by reducing 5-LO, IL-6 and TNF-*α*, and improves cognitive function and cerebral microvesselar integrity	*in vivo*	70

	Chrysophanol	Increases the viability of neuronal cells, inhibits apoptosis via increasing Bcl-2 expression and decreasing the expression of Bax and AIF; reduces ROS levels and prevents mitochondrial fission by suppressing the dephosphorylation of Drp1	*in vitro*	71

	Rhein lysinate	Reduces APP by improving the expression of SIRT1, decreases the expressions of TNF-*α* and IL-6, reduces ROS levels and increases the levels of GSH-px and SOD	*in vivo*	72

	Rhein-huprine hybrids	Alleviates the A*β*-induced synaptic dysfunction, increases the content of synaptic proteins and induces LTP; reduces the levels of A*β*, improves mature APP levels, suppresses the activities of AChE, BuChE and BACE-1, and reduces the aggregation of A*β*	*in vivo *and *in vitro*	73

	Rhein-huprine hybrids	Decreases memory disorders and A*β* levels, reduces tau phosphorylation and brain inflammation, and induces LTP	*in vivo *	75

	Rhein-huprine hybrids	Suppresses the activities of AChE and BACE-1 via reducing oxidative stress and A*β*/tau aggregation	*in vitro*	76

	Tacrine-rhein hybrids	Inhibits AChE-induced A*β* aggregation, and shows metal-chelating activity with less side effects	*in vivo *and* in vitro *	74

	Danthron	Inhibits neuronal injury, and suppresses oxidative damage via reducing membrane lipid peroxidation	*in vitro *	65

Depression	Emodin	Improves behavioral performance, reduces serum corticosterone level, increases the expressions of BDNF and GR, and improves the anhedonia symptom by increasing sucrose consumption	*in vivo*	81

	Chrysophanol	Alleviates inflammation via reducing the levels of IL-1*β*/6 and TNF-*α*, and attenuates the expression of P2X7/NF-*κ*B pathway related proteins inculding P2X7, p-IKK*α*, p-IKK*β*, p-I*κ*B*α* and p-NF-*κ*Bp65	*in vivo*	82

Epilepsy	Emodin	Restores behavioural disorders and abnormal EEG changes by attenuating the expressions of MDR1, COX-2, NMDA receptor and P-glycoprotein	*in vivo*	83

Neuropathic pain	Emodin	Alleviates the hyperalgesia via reducing the expression of P2X_2/3_ receptor	*in vivo*	88

Diabetic encephalopa-thy	Chrysophanol	Improves cognition function by decreasing the expressions of IL-1*β*/4/6 and TNF-*α*, and reduces nerve cell death via inhibiting astrocyte activity	*in vivo*	90

Lead poisoning	Chrysophanol	Reduces learning and memory damage, improves mitochondria and rough endoplasmic reticulum injury, raises SOD and GSH-Px activities, and decreases MDA level	*in vivo*	91

Encephalitis	Rheum palmatum methanol extract	kills JEV, and inhibits JEV yields and infectivity	*in vitro*	92
